# Genetic Diagnosis of Pyruvate Kinase Deficiency in Undiagnosed Iranian Patients with Severe Hemolytic Anemia, using Whole Exome Sequencing

**DOI:** 10.34172/aim.2022.108

**Published:** 2022-10-01

**Authors:** Jafar Mehrabi Sisakht, Maghsood Mehri, Hossein Najmabadi, Azita Azarkeivan, Maryam Neishabury

**Affiliations:** ^1^Genetics Research Center, University of Social Welfare and Rehabilitation Sciences, Tehran, Iran; ^2^Kariminejad-Najmabadi Pathology & Genetics Centre, Tehran, Iran; ^3^Blood Transfusion Research Center, High Institute for Research and Education in Transfusion Medicine, Tehran, Iran

**Keywords:** Genetic diagnosis, PKLR gene, Pyruvate kinase deficiency, Whole Exome Sequencing

## Abstract

**Background:**

After ruling out the most common causes of severe hemolytic anemia by routine diagnostic tests, certain patients remain without a diagnosis. The aim of this study was to elucidate the genetic cause of the disease in these patients using next generation sequencing (NGS).

**Methods:**

Four unrelated Iranian families including six blood transfusion dependent cases and their parents were referred to us from a specialist center in Tehran. There was no previous history of anemia in the families and the parents had no abnormal hematological presentations. All probands presented severe congenital hemolytic anemia, neonatal jaundice and splenomegaly. Common causes of hemolytic anemia were ruled out prior to this investigation in these patients and they had no diagnosis. Whole exome sequencing (WES) was performed in the probands and the results were confirmed by Sanger sequencing and subsequent family studies.

**Results:**

We identified five variants in the *PKLR* gene, including a novel unpublished frameshift in these families. These variants were predicted as pathogenic according to the ACMG guidelines by Intervar and/or Varsome prediction tools. Subsequent family studies by Sanger sequencing supported the diagnosis of pyruvate kinase deficiency (PKD) in six affected individuals and the carrier status of disease in their parents.

**Conclusion:**

These findings show that PKD is among the rare blood disorders that could remain undiagnosed or even ruled out in Iranian population without performing NGS. This could be due to pitfalls in clinical, hematological or biochemical approaches in diagnosing PKD. Furthermore, genotyping PKD patients in Iran could reveal novel mutations in the *PKLR* gene.

## Introduction

 In our previous studies, whole exome sequencing (WES) helped us to identify various rare types of hereditary blood disorders among Iranian individuals with severe anemia and no diagnosis. These included sideroblastic anemia in four families,^1^ Diamond-Blackfan anemia, adenosine deaminase deficiency and congenital dyserythropoietic anemia in other group of four families^2^ and a case of BENTA disease (B cell expansion with NF-κB and T cell anergy) in one family.^3^

 Here, we present the diagnosis of pyruvate kinase deficiency (PKD) by WES in another group of four Iranian families in whom the probands had severe hemolytic anemia and the common causes of this phenotype, including hemoglobinopathies, G6PD deficiency, spherocytic and autoimmune hemolytic anemia^4^ were ruled out prior to this investigation.

 With an estimated frequency of 3–8 in 1 000 000,^5^ PKD is defined as a rare but the most common defect in the glycolytic pathway. This disease is associated with congenital non-spherocytic hemolytic anemia, with a variety of clinical manifestations and severity, ranging from fetal hydrops to fully compensated anemia.^5-9^ Carrier frequencies of 1-5% have been reported in two provinces in Iran.^10^

 At least 371 pathogenic mutations in the *PKLR *gene have been associated with PKD^8,11^ and at least 600 affected families have been reported.^9^ Meanwhile, it is believed that many of the PKD cases remain undiagnosed or misdiagnosed and the frequency of the disease could be underestimated.^9,12^ Pitfalls in the diagnosis of PKD are due to several reasons. These include its overlapping clinical phenotypes with other types of hemolytic anemia, the autosomal recessive nature of the disease (where parents are normal with no history of disease in the family), wide variability in disease severity (where some patients remain unaware of their disorder) and error proneness of PK enzymatic activity assays. The latter is further complicated when the patients receive RBC transfusion. Finally, unfamiliarity with the disease even among specialists also contributes to undiagnosis of this disease.^5,9^ With the aim of improving and harmonizing the diagnosis of PKD, a global international working group, consisting of 20 different centers, has published diagnostic recommendations, which is endorsed by the European Network in Rare Hematological Diseases. The recommended guideline relies on complementary techniques including biochemical analysis and next generation sequencing (NGS), which are performed in different order in different laboratories.^9,12^

 Currently, there is no definite cure for PKD and supportive care for this disease include transfusions, splenectomy and chelation therapy with essential need for monitoring to avoid complications. Meanwhile, ongoing research in PK activators^13,14^ and gene therapy^15^ provide some hope as potential future treatment strategies. In parallel, efforts for optimizing diagnostics are also proceeding.^7,9,11^

 Here, we report identification of pathogenic *PKLR* variants in four Iranian families with severe hemolytic anemia in undiagnosed probands, by WES.

## Materials and Methods

**Figure 1:**
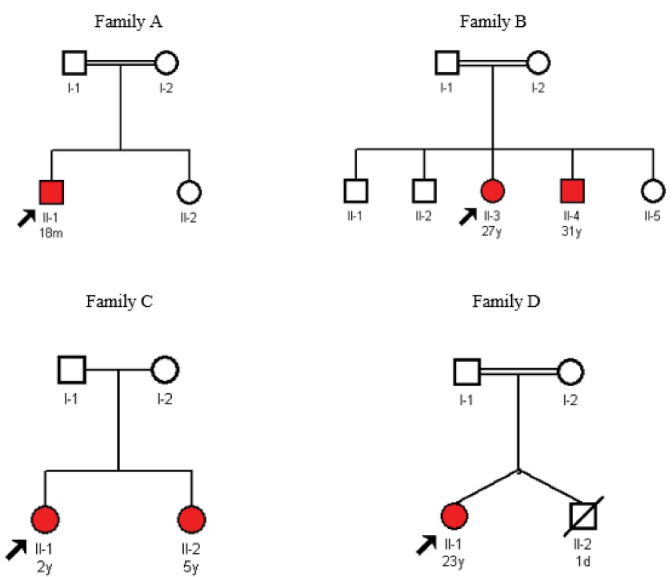
Pedigrees for Families A-D.

 Four unrelated Iranian families (Figure 1, families A, B, C & D) including six affected individuals (three children and three adults) together with their unaffected parents (eight individuals) were referred to our research center from an adult thalassemia clinic in Tehran. All probands presented severe jaundice and anemia in early days of their life and received exchange transfusion. They were red blood cell (RBC) transfusion dependent at regular intervals since infancy and either presented splenomegaly or were splenectomized. Hemoglobinopathies, G6PD deficiency, spherocytosis and autoimmune hemolytic anemia were ruled out in all families prior to this investigation. In addition, biochemical assay of pyruvate kinase (PK) enzyme activity in the probands of the first and third family (Figure 1A; Ⅱ-1 and Figure 1C; Ⅱ-1 respectively) had shown normal results prior to this study. No family history of anemia was present in these families and parents in these families had normal hematological indices. More specific data including demographic information related to the affected individuals in each family were as follows (unaffected siblings did not participate in this study):

*Family A:*The proband (Figure 1A; Ⅱ-1) (from the Kohgiluyeh and Boyer Ahmad province) was an 18-month-old boy. He had been admitted to the neonatal ward due to jaundice and laboratory findings of hemolytic anemia on the 1^st^ day of his life. He had received exchange transfusion on the 2^nd^ day of his life and had developed polar and splenomegaly 14 days later. He has received RBC transfusion every 20–25 days since his first exchange transfusion. His bone marrow examination revealed essentially cellular marrow, erythroid hyperplasia, mild megaloblastic changes and less than 1% erythroid binucleation. Other abnormal hematological findings included moderate normochromic anemia and mild anisocytosis, nucleated red blood cells (1–2/100 WBCs); low RBC (3.56×10^6/µL); low hemoglobin (8.7 g/dL), low hematocrit (26.3%), low mean corpuscular hemoglobin (MCH) (24.4 pg), high platelet (650×10^3/µL) and high reticulocyte (12%) indices. He also had increased SGOT (serum glutamic-oxaloacetic transaminase) and bilirubin levels. The biochemical assay of enzyme activity performed prior to this investigation was reported normal in this proband. His parents were first cousins and had normal hematological indices.

*Family B:*The proband in the second family (Figure 1B; Ⅱ-3) was a 27-year-old female (from the Fars province) with splenomegaly and RBC transfusion intervals of every 22–23 days. She had a 31-year-old affected brother (Figure 1B; Ⅱ-4), who was splenectomized at 9 years of age. Splenectomy had reduced his blood transfusion intervals from the regular interval of every 23 days to irregular intervals of 60-80 days. They both had presented severe jaundice and splenomegaly at infancy and received exchange transfusion in the first days of their life. The results of childhood bone marrow examination were not recorded and available for any of these siblings. Abnormal hematological findings in the proband included low RBC (3.2×10^6/µL), low hemoglobin (9.2 g/dL), low hematocrit (26.9%) and high reticulocyte (5.7%). She had a normal platelet count. The abnormal hematological indices of the proband’s affected brother, who was splenectomized included high WBC (14.99×10^3/µL), low RBC (2.81×10^6/µL), low hemoglobin (9.1 g/dL), low hematocrit (29.3%), high MCV (104.3 fL), high platelet (810×10^3/µL), high nucleated red blood cells (12/100 WBCs), polychromasia (3+), anisocytosis (3+) and macrocytosis (slight). Both affected siblings showed increased SGOT, SGPT (serum glutamic-pyruvic transaminase) and bilirubin. Their parents were second cousins and had normal hematological indices.

*Family C:*The proband in the third family (Figure 1C; Ⅱ-1) was a 2-year-old girl (from the Khuzestan province) with an affected 5-year-old sister (Figure 1C; Ⅱ-2). They both had presented severe jaundice and anemia at birth and received exchange transfusion in infancy. They both had RBC transfusion intervals of every 30 days since their exchange transfusion. Bone marrow examination of the elder sister, who presented splenomegaly, showed normocellular marrow with increased erythroid series. Their abnormal hematological findings included low RBC (2.16×10^6/µL), low hemoglobin (5.8 g/dL), low hematocrit (18.0%) and high platelet values (600×10^3/µL) in the proband and low RBC (2.62 x10^6/µL); low hemoglobin (7.3 g/dL) and low hematocrit (21.3%) in her affected sister. Increased SGPT and bilirubin were reported in the elder sister of the proband. The parents in this family were non-consanguineous and had normal hematological indices.

*Family D:*The proband in the last family (Figure 1D; Ⅱ-1) was a 23-year-old young woman (from the Khorasan province). She had a twin brother, who had died at first days of his life. She received RBC transfusion with 40–50 days intervals since her first exchange transfusion at infancy. She had been splenectomized at 6 years of age, with no great improvement in her blood transfusion needs. Her bone marrow examination showed normocellular marrow with erythroid lineage hyperplasia. Other abnormal hematological findings were as follows: low RBC (3.05), low hemoglobin (9.5), low hematocrit (30.2), low mean corpuscular hemoglobin concentration (MCHC) (31.5(g/dL), high platelet (760×10^3/µL), high reticulocyte (7.3%), nucleated RBC (1/100WBCs), increased bilirubin, SGOT, SGTP and alkaline phosphatase, anisocytosis (2+), poikilocytosis (1+), macrocytosis (1+) and Howell-Jolly body (1+). Her parents were first cousins and had normal hematological indices.

###  Whole Exome Sequencing

 After informed consent, 5–10 mL blood samples were taken from the probands, their parents and their affected siblings. The salting out procedure^16^ was used to extract DNA. After qualitative and quantitative evaluation using standard techniques, the probands’ DNA was subjected to WES (HISeq400 sequencer, Sure Select V6-Post by Macrogen Inc.) Mapping reference was hg19 from UCSC (original GRCh37 from NCBI, Feb. 2009). The red cell NGS gene panel list (http://www.viapath.co.uk/our-tests/red-cell-gene-panel) was used to analyze the data. The UCSC genome browser, Intervar^17^ and Varsome^18^ web tools were used for bioinformatic predictions of the pathogenicity of the variants. The population-specific Iranome database ^19^ was used to investigate the frequency of the variants in the Iranian population.

###  PCR and Sanger Sequencing

 PCR reverse (R) and forward (F) primers (Table 1) were designed by primer 3 to amplify the variant region in the DNA of the probands and their family members. Sanger sequencing was performed to sequence the variant region in each individual.

**Table 1:** PCR Primers for Sanger Sequencing

**Family**	**Primer Name**	**Primer Sequence (5'->3')**	**Length (bp) **	**Product Length **
A	*PKLR*-EXON10-F	GCCCAGAGAAGTATGATGACTTAC	24	472
	*PKLR*-EXON10-R	GTGATATGCCAGACTGATATCTCAG	25	
B	*PKLR*-EXON2-F	ATCCTAGCTGATCCATACTTAG	22	504
	*PKLR*-EXON2-R	GCACCTCAAGAAATACCAATAG	22	
C	*PKLR*-EXON1-F	CCAAAACCCACCTAGCCAGT	20	550
	*PKLR*-EXON1-R	GCTCCCTGGATTCACTAGAGC	21	
	*PKLR*-EXON2-F	CCTAGATTTGAATCCTAGCTGATCC	25	757
	*PKLR*-EXON2-R	CCCGGCCTCACTTTCTAAC	19	
D	*PKLR*-EXON5-F	GGGAAGGTGTGATCGGTCTG	20	537
	*PKLR*-EXON5-R	TACCATGCTGAGTCCATCGC	20	

## Results

###  Whole Exome Sequencing and Family Study by Sanger Sequencing

**Figure 2:**
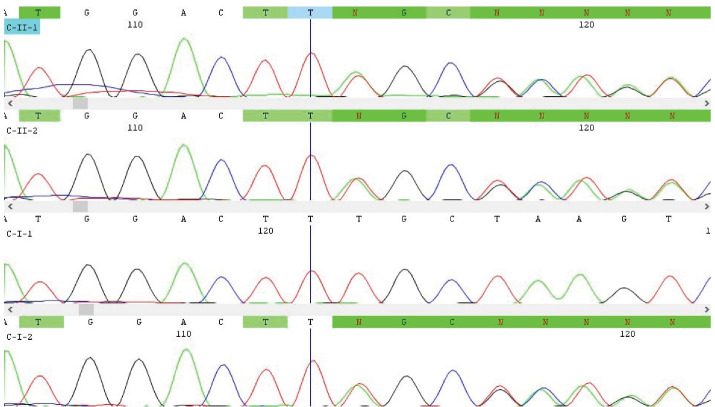
Chromatograms of Novel Frameshift *PKLR *Variant c.72_73insCT: p.K25Lfs*5 in family C. As shown, the proband (C-Ⅱ-1) and her affected sister (C-Ⅱ-2) inherited this variant from their carrier mother (C-Ⅰ-2).

 Five variants in the *PKLR* gene, including one previously unpublished frame shift variant, was identified by WES. Family studies by Sanger sequencing showed variants listed in Table 2 in homozygous (families A, B and D) or compound heterozygous state (family C) in affected individuals. Parents in each family were heterozygous carriers. Chromatograms are only presented for the novel variant identified in family C (Figure 2).

**Table 2:** Variants Identified by Whole Exome Sequencing in Probands of Families A-D

**Family**	**A**	**B**	**C**		**D**
Probands and other affected member homozygous for the variant	II-1	II-3 and II-4	II-1 and II-2	II-1 and II-2	II-1
Variant coordinate	1: 155261637	1: 155270000	1:155271114	1:155269889	1: 155265081
HGMD variant	CM981581	CM1615476	-	Different nucleotide substitution in the same position as CM984072 (GGG>AGG, Gly95Arg)	CM981554
Ref>Alt	G>A	G>A	T >TAG	C>A	C>A
Variant Type	stop gain	stop gain	frameshift insertion	Missense variant	stop gain
Zygocity	Hom	Hom	Het	Het	Hom
HGVS	*PKLR*: NM_000298.6:exon10:c.1528C>T: p.R510X	*PKLR*: NM_000298.6:exon2:c.172C>T: p.Q58X	*PKLR*:NM_000298:exon1:c.72_73insCT: p.K25Lfs*5	*PKLR*: NM_000298.6:exon2:c.283G>T: p.G95W	*PKLR*: NM_000298:exon5:c.520G>T: p.E174X
dbSNP-ID	rs1331742633	-	-	rs750857114	-
InterVar prediction	Pathogenic (PVS1, PM2, PP3)	Likely pathogenic (PVS1, PM2)	Pathogenic (PVS1, PM2,PP2)	Uncertain significance (PM1, PM2, PP3)	Pathogenic PVS1, PP3, PM2
Varsome	Pathogenic (PVS1, PS3, PM2, PP3,PP5)	Pathogenic (PVS1, PM2, PP3)	Likely pathogenic (PVS1, PM2)	Pathogenic PVS1, PM5, PM2, PP2, PP3	Pathogenic PVS1, PP3, PM2
MAF in Iranome	Not Reported	Not Reported	Not reported	Not reported	Not Reported
MAF in gnomAD Total (Exomes/Genomes)	0.00001414	Not reported	Not reported	0.000003986	Not reported
CADD_phred score	44	34	-	34	35
Publications	11, 20, 21	11, 22	-	21	21

####  Variant in Family A

 The 18-month-old proband in the first family (Figure 1A; Ⅱ-1), had a previously reported stop gain variant c.1528C>T:p.R510X^11,20,21^ (Table 2). This variant is located just next to the c.1529G>A; p.R510Q, which is reported as the most frequent mutation of the *PKLR* gene in Northern Europe and USA.^5^ This variant was predicted as pathogenic by both Intervar and Varsome.

####  Variant in Family B

 In the second family, the 27 year-old proband (Figure 1B; II-3) and her 31 year-old affected brother (Figure 1B; Ⅱ-4), had a stop gain variant c.172C>T:p.Q58X (Table 2), which was previously submitted to the Leiden Open Variation Database (LOVD) as an unpublished observation from Iran^11^ and a second observation of this variant was recently reported in PKD patients.^22^ This variant was predicted as likely pathogenic in Intervar and as pathogenic in Varsome.

####  Variants in Family C

 Theaffected siblings with 2 and 5 years of age in the third family (Figure 1C; Ⅱ-1 and Ⅱ-2) had two variants. These were c.72_73insCT:p.K25Lfs*5 (Table 2; Figure 2) and c.283G>T:p.G95W (Table 2) in compound heterozygous state. The first frameshift variant, which was predicted as pathogenic by Intervar, was novel and not published before. The second missense variant, which was previously published as a causative variant for PKD,^21^ was annotated as a variant of unknown significance and pathogenic by Intervar and Varsome, respectively.

####  Variant in Family D

 In the last family, a previously reported variant c.520G>T:p.E174X^21^ (Table 2) was identified in the 23-year-old proband (Figure 1D; Ⅱ-1). This variant was predicted as pathogenic by both Intervar and Varsome prediction tools.

## Discussion

 Six undiagnosed patients, including three children and three adults from four unrelated Iranian families were referred to our research center. These patients presented the typical clinical and hematological manifestations of congenital hemolytic anemia with unidentified reason. Initial suspicion to common causes of hemolytic anemia, including alpha and beta thalassemia, G6PD deficiency and autoimmunity^4^ was eliminated prior to this investigation. In addition, biochemical analysis for pyruvate kinase enzyme activity prior to our investigation had shown normal results for the probands in families A and C.

 After performing WES in the probands, we identified five variants in *PKLR* gene, which were predicted as pathogenic according to ACMG guidelines by Intervar and/or Varsome web servers. These included one unpublished new *PKLR* frameshift variant (c.72_73insCT:p.K25Lfs*5), which was identified in two affected children of family C in compound heterozygous state, together with a previously reported *PKLR* variant (Table 2). The WES findings (Table 2) and segregation analysis (Only shown for novel variant; Figure 2) in all families could conclude diagnosis of PKD in affected families and showed that the results of previously performed biochemical enzyme activity assay in two of these families were false normal.

 The result of this investigation places PKD among other rare blood disorders that could remain undiagnosed in the Iranian population without WES.^1-3^ In addition, findings in this study raise serious concerns on the risk of false normal results in biochemical analysis of PK enzyme activity in diagnostic setups. Some possible reasons for false normal results include contamination with donor blood in blood transfusion dependent affected individuals, incomplete removal of platelets and leucocytes during sample preparation and increased reticulocytes in PKD patients, which express more active isozymes to rescue glycolysis.^9^

 Diagnostic guidelines, endorsed by the European Network in Rare Hematological Diseases, which were published in 2019, have recommended strategies for accurate diagnosis of PKD and reducing the errors.^9^ According to these guidelines, the PK activity assay by spectrophotometry described by Beutler in 1984^23^ is recommended as a reference test for biochemical enzymatic assay. Correct timing of this test, 120 days after the last transfusion (as the optimal time distance) is important in transfusion dependent patients. Moreover, testing the parents, who would be expected to show low enzyme activity if they are carriers, is recommended to prevent false normal results related to blood transfusion in their affected children. For optimum RBC purification, alpha cellulose/microcrystalline column is recommended with considering buffy coat removal as an alternative. To minimize false normal results due to reticulocyte interference in PKD patients, the use of a control sample from a patient with the same degree of reticulocytosis and/or comparing the PK activity to other cell-age dependent enzymes of the patient such as hexokinase is recommended.^5-9^ A rarer reason for false normal results is a kinetically abnormal mutant PK enzyme that behaves normal in laboratory conditions but is ineffective *in vivo*. Thermal stability test could reveal the true functional effect of such mutations.^7,9^

 Although compared to difficult to perform and error prone biochemical assays, NGS is quick and accurate, it has its own limitations. First of all, it is still not affordable for many patients and secondly, if a variant of unknown significance is identified in *PKLR* that cannot be predicted as pathogenic based on ACMG guidelines by reliable prediction databases, its pathogenicity has to be confirmed by PK enzymatic assay. Therefore, both enzyme analysis and DNA studies are recommended as complementary techniques for the diagnosis of PK deficiency.^5,9^

 Application of WES as a new diagnostic approach in diagnosing rare types of anemia in Iran helped us to find the pathogenic mutations in the *PKLR* gene, including a novel variant in severe hemolytic anemia patients without diagnosis. Subsequent data analysis and family investigation confirmed the diagnosis of PKD in these individuals. This approach could lead to the diagnosis of more PKD patients as well as identifying more novel mutations in the *PKLR* gene.

 In conclusion, PKD is among the rare types of hereditary blood disorder that could remain undiagnosed in Iran. This reflects the complexity of its clinical, hematological and biochemical diagnostics that need regular evaluation and update in diagnostic centers. Meanwhile, WES remains a quick and accurate way to diagnose rare types of hereditary blood disorders, including PKD among undiagnosed individuals with severe anemia. Furthermore, genotyping PKD patients in Iran could reveal novel mutations in the *PKLR* gene.
